# Evaluation of the nutritional status of infants from mothers tested positive to HIV/AIDS in the health district of Dschang, Cameroon

**DOI:** 10.11604/pamj.2014.18.91.2794

**Published:** 2014-05-26

**Authors:** Martin Sanou Sobze, Raoul Guetiya Wadoum, Edith Temgoua, Jean-Hubert Donfack, Lucia Ercoli, Ersilia Buonomo, Joseph Fokam, Bruna Djeunang Dongho, James-Francis Onohiol, Yannick Zefack, François Ngoufack Zambou, Alberto Cresci, Gianluca Russo, Vittorio Colizzi

**Affiliations:** 1 Department of Biomedical Sciences, Faculty of Sciences, University of Dschang, Dschang, Cameroon; 2 Department of Biochemistry, University of Dschang, Dschang, Cameroon; 3 Department of Biology, University of Rome Tor Vergata, Roma, Italia; 4 Chantal Biya International Reference Centre (CIRCB) for research on HIV/AIDS prevention and management, Yaoundé, Cameroon; 5 Soledad Medical Institute (IMES); 6 Department of public health of the University of Rome Tor Vergata, Roma, Italia; 7 Department of comparative Biochemistry and morphological science, University of Camerino, Camerino, Italia; 8 Department of tropical and infectious disease, University of Rome La Sapienza, Roma, Italia

**Keywords:** Nutritional status, HIV/AIDS, infants, Body Mass Index

## Abstract

**Introduction:**

Poor infant feeding practices are common in Africa, resulting in physical and intellectual developmental impairments. Good feeding practices are crucial, especially in the first year of growth. HIV/AIDS has worsened the clinical and nutritional status of both mothers and their children, exacerbating high rates of malnutrition. The aim of this study was to assess by participative approach, the nutritional status of infants from mothers tested positive to HIV in the health district of Dschang.

**Methods:**

This is a cross sectional study with a period of recruitment of 2 years (2010-2012). Data Collection was done by the aim of a personal slip followed by training to strengthen the nutritional and hygienic capacity of targeted parents. Height and weight of infants were measured and body mass index (BMI) calculated.

**Results:**

Significant difference (p ≤ 0.05) was noticed in height-for-age z-score (HAZ) of girls aged between 1 to 2 years compared to 1-year old girls as well as to boys of all ages, defining them as stunted. Furthermore, the weight-for-age z-score (WAZ) results indicate that both girls and boys of all age are in moderate state of malnutrition. The results of BMI thinness classified according to gender and age groups, indicates that most infants (68/130, 52.3%) showed grade 2 thinness predominantly in 2-years old both boys and girls. However, no participants fall within the normal category for age and sex, as well as overweight and obesity categories.

**Conclusion:**

Undernutrition exists among infants from mothers tested positive to HIV residing in Dschang, as most of the infants are underweight, and malnourished.

## Introduction

Breastfeeding is a pillar for child survival; it reduces morbidity and mortality in children worldwide [1]. However, since the early 1980s when it was discovered that HIV could be transmitted to infants through human milk, the healthfulness of breastfeeding has been questioned, because of the risk of mother-to-child transmission of HIV (MTCT) [2]. Indeed, scientists, policymakers, and program managers have spent the last several decades struggling to characterize the proportion of risk of MTCT attributable to breast milk and to develop appropriate and feasible guidelines on infant feeding in settings where HIV is endemic [3–6]. For this reason, it has been said that the HIV pandemic has threatened to “knock breastfeeding off its pedestal as a pillar of child survival” [7].

Infancy and early childhood are periods of particular risk for growth failure and undernutrition [8–10]. The risk for infants from HIV-positive mothers to be undernourished is accentuated by the prevention of transmission of the HIV virus to the newborn since exclusive breastfeeding have to be stop after 6 months [11]. Therefore, assessment of growth and nutritional status by objective anthropometric methods (weight, length, head circumference, body mass index, skinfold thicknesses, and midupper arm circumference) is central to the identification of growth failure and undernutrition [12].

There are several studies [13–17], investigating the problem of undernutrition among infants in different parts of Africa and in Cameroon. However, little information exists regarding malnutrition among infants from mothers tested positive to HIV/AIDS residing in Dschang-Cameroon.

The present observational study evaluates the nutritional status of infants from HIV-positive mothers in the health district of Dschang-Cameroon. We thought that such data are essentially important for public health standpoint as they would provide reliable bases for instituting appropriate strategies to identify and combat factors associated with nutritional abnormalities in infants from HIV-positive mothers as well as achieving the “3-0” call of the UNAIDS to ensure zero new infection, zero discrimination, and zero mortality associated to HIV/AIDS by 2015 [18].

## Methods

### Study area

**Figure 1:**
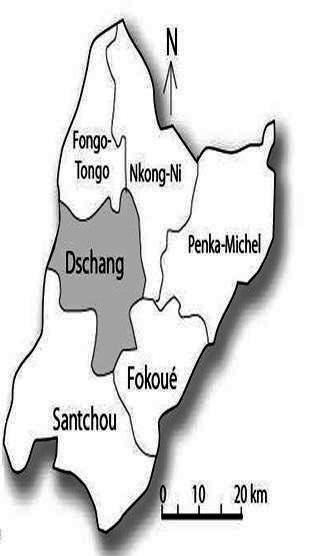
Height measurement [31]

The Dschang health district is located in the West region of Cameroon (Figure 1) with a population of 211,818 inhabitants, of which 38,127 (18%) are children under 5-years old; the district is divided in 22 areas. The population of the health district of Dschang is dynamic and their principal activities are agriculture, trade and breeding. According to a field study realized by the students of the University of Dschang, the accessibility to drinking water is very limited and the electrification is very unequally distributed.

The health district is crossed by a principal road on which is connected up with many minor roads which serve the villages. The relief is generally very accidental, and consists of an alternation of hills and valleys. The most encountered health problems are: Malaria, AIDS, malnutrition, Hypertension and obesity. Moreover, the presence of the University within the health district of Dschang gives him a particular configuration.

### Sample

This is a cross sectional study with a period of recruitment of 2 years (2010-2012). The target samples were infants (HIV negative) from mothers tested positive to HIV/AIDS. Data Collection was done by the aid of a personal slip followed by training to strengthen the nutritional and hygienic capacity of targeted parents. Height and weight of 130 infants (72 boys and 58 girls) aged 1 to 3 years were measured and the body mass index (BMI) calculated. A two-stage probability sampling method was used. The first stage includes the selection of mothers, and the second stage was the selection of infants from chosen mothers based on their vulnerability.

### Anthropometric measures

**Figure 2:**
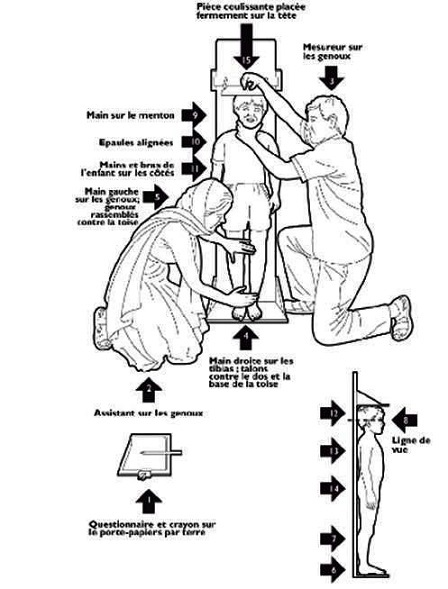
Location of the Dschang town in the Menoua Division-West region-Cameroon [32]

Height and weight were measured according to standard anthropometric methods (International Society for the Advancement of Kinanthropometry: ISAK) [19]. Height was measured to the nearest 0.1 centimeters (cm) with a measuring tape (Seca) as well as a bare feet with participants standing upright against a mounted stadiometer (Seca) (Figure 2). Weight was measured to the nearest 0.1 kilogram (kg) with participants lightly dressed (underwear and T-shirt) using a baby scale (Seca-Säuglingwage) as well as the Health Scale - Mic balance. BMI was computed as weight / (height) 2 (kg m-2). The investigators themselves collected all the data and took care of inter-rater bias. The instruments used in the process of data collection like weighing machines were the same for all data collection. Weighing scale was calibrated on daily basis.

### Statistical analysis

Means and standard deviations were calculated for body mass, stature and BMI across sex and age groups. Differences in the mean body mass, stature and BMI were evaluated for boys and girls according to age-group, using an independent samples t-test. The z-scores of < -2.0 was calculated to derive HAZ and WAZ category of stunt and underweight, respectively. The children were classified on the basis of WHO 2007 BMI thinness classification [20]. Children with BMI of 17.0-18.5, 16.0-17.0 and < 16.00 were classified as grade 1, 2 and 3 thinness respectively. Children with a BMI of 18.5-25.0, 25.0- < 30 and > 30 were categorized as normal, overweight and obese, respectively. All statistical analyses were performed using Statistical Package for Social Sciences (SPSS) version 17.0. A probability level of ≤ 0.05 was considered to be statistically significant.

### Ethical approval

Authorization for this study was obtained from the authorities of both hospitals and ethical clearance from the ethical committee of the Cameroon Ministry of Public Health.

## Results

One hundred and thirty HIV negative infants (72 male and 58 female), aged from 12 to 36 months, born from HIV-positive mothers were included in this study. Overall, the mean values for body weight (Boys: 9.2 ± 2.4 kg; Girls: 9.47 ± 4.1 kg), stature (Boys: 76.25 ± 7.7 cm; Girls: 76.69 ± 6.1 cm) and BMI (Boys: 16 ± 1.8 kg.m-2; Girls: 16.20± 1.1 kg.m-2), were not significantly different (p ≤0.05) neither in girls nor in boys (Table 1). Significant sex difference for body mass index, weight and height were not observed.

**Table 1:** Characteristics of the participants

			Weight (kg)		Height(cm)		BMI(kg.m^-2^)	
Age	Boys	Girls	Boys	Girls	Boys	Girls	Boys	Girls
(years)	n	n	(SD)	(SD)	(SD)	(SD)	(SD)	(SD)
1	22	17	6.5 (2.8) *	6.8 (3.2) *	65.0 (2.2)	68.4 (3.7)	15.38 (1.2)	14.53 (1.3)
2	31	25	10.2 (1.5)	10.6 (4.5)	78.1 (2.2)*	77.3(2.5)*	16.72 (1.7)	17.74 (2.2)
3	19	16	11.5 (2.5)	11.0 (4.4)	85.1 (3.6)	83.0 (4.2)	15.88 (2.1)*	15.97 (1.9) *

* Statistically not significant (p ≥ 0.05) in the line for each sex and parameter; kg: kilograms; Kg.m-2: kilograms per square meter; SD = standard deviations; n = number of participants

However, significant differences (p ≤ 0.05) were noticed in height-for-age z-score (HAZ) of girls aged between 3 to 2 years compare to the value obtained with 1 year old girls as well as in boys of all ages. Furthermore, the weight-for-age z-score (WAZ) results indicate that both girls and boys have normal value of WAZ (Table 2).

**Table 2:** Anthropometric indices of nutritional status of children

		Height-for-ages z-score(HAZ)	Weight-for-age z-score ( WAZ)
Age (years)	n	Mean (SD)	Mean (SD)
**Boys**			
1	22	-0.45 ( 0.12)	-0.54 (0.13)
2	31	-0.50 (0.20)	-0.47 (0.11)
3	19	-0.31 (0.15)	-0.20 ( 0.10) *
**Girls**			
1	17	-0.91 (0.35) *	-0.10 (0.05)
2	25	-2.92 (0.24)	-0.08 (0.03) *
3	16	-2.0 (0.31)	-0.11 ( 0.02)

* Statistically significant (p < 0.05) in the column for each gender; n = number of participants; SD = standard deviation

The largest percentage of the infants were not stunted (89; 68.46%) HAZ and (130; 100%) WAZ and 31.5% (41) of the children was moderately stunted (Table 3).

**Table 3:** Nutritional status of children using the Height-for-age Z-scores (HAZ) and Weight-for-Age Z-scores (WAZ)

	HAZ	WAZ
Nutritional status	n (%)	n (%)
Severe malnutrition	-	-
Moderate (stunting)	41 (31.54)	-
Normal ( Not stunting)	89 (68.46)	130 (100)

**WAZ** = Weight-for-age Z-score; **HAZ** = Height-for-age Z-score; % = percentage

Results of BMI thinness classification of the participants according to gender and age groups (Table 4), indicate that age variations exist in the different BMI thinness classifications. The majority of the children (68) exhibited Grade 2 thinness, which was predominant in both boys and girls of 2 years of ages. Gender wise, 30 boys and 27 girls fell within Grade 3 thinness category and only girls (n = 5) were in the Grade 1 thinness category. Furthermore, 42 boys and 26 girls showed Grade 2 thinness. However, no participants fell within the normal category. Overweight and obesity was absent in boys and girls.

**Table 4:** BMI thinness classification of children according to gender

BMI thinness classification*						
**Age**	< 16.00	16.0-17.0	17.0-18.5	18.5-25.0	25.0- < 30	> 30
**(years)**	**Grade 3 thinness**	**Grade 2 thinness**	**Grade 1 thinness**	**Normal**	**Overweight**	**Obesity**
**Boys**						
1	17	5	0	0	0	0
2	0	31	0	0	0	0
3	13	6	0	0	0	0
**Girls**						
1	17	0	0	0	0	0
2	0	20	5	0	0	0
3	10	6	0	0	0	0

* WHO 2007 BMI thinness classification

## Discussion

In developing countries, malnutrition in infants is a public health concern. This study evaluates the nutritional status of HIV negative infants born from mothers tested positive to HIV/AIDS in the health district of Dschang-Cameroon. Nutritional status is an integral component of the overall health of an individual [21], and provides an indicator of the well-being of infants living in a particular region [22].

The findings of the present study reflect a seemingly high prevalence of malnutrition (31.54%) among infants. Underweight is used as a composite indicator to reflect both acute and chronic undernutrition, although it cannot distinguish between them [23]. Furthermore, these results indicated that boys are more underweight than girls.

Using WHO 2007 BMI thinness classification, we noticed that age variations exist in the different BMI thinness classification. While majority of the children exhibited Grade 2 thinness (n = 68), which was predominant at all ages in both boys and girls, overweight and obesity were absent in all infants.

Paediatric HIV infection is associated with growth failure in most settings, even in infants receiving antiretroviral drugs [24–26]. Reason while, more effort is made nowadays to restrict the transmission of the HIV virus to infants by their mother. The WHO recommend that in other to reduce the contamination of infants, exclusive breastfeeding have to be substituted by artificial feeding from the sixth month because the viral content increase in breast milk and can easily infect the baby [27, 28]. This implied that parents have to spend enough money in other to satisfy the nutritional needs of the infants [29]. Unfortunately, most of the parents involved or infected are in developing countries and have low income and cannot provide all the essentials nutrients required to provide a good nutritional status to infants [30]. Moreover, most of the HIV-positive parents are generally stigmatized, thus making the infants more vulnerable to undernutrition.

Nevertheless, parents receive support from locally based non-governmental organizations (NGO) such as “PIPAD” and it's Italian partners devoted to the promotion of health and welfare. This assistance consists to provide foods and essentials nutrients as well as training HIV positive parents in other to strengthen their nutritional and hygienic capacity and manage self incomes activities. Furthermore, this appalling nutritional situation among our sample can be linked to ignorance and therefore Cameroon government's nutritional intervention programs have to be revised and strengthen.

## Conclusion

There is malnutrition among infants (HIV negative) from mothers tested positive to HIV/AIDS residing in Dschang-Cameroon as most of the infants are undernourished. As the quality of future human resources depends on the present day infants, improvement of the nutritional level of today's infants should be given top priority. Thus, there is a need to emphasize on infant feeding practices and key messages have to be carried to the general population, in particular to family living with HIV/AIDS. In addition, to reduce the vertical transmission, promote the development and growth of infants from parents tested positive to HIV/AIDS a regular, well oriented and tight follow up for feeding options should be adopted.
